# Supplementary material for: Health promotion interventions for the control of hypertension in Africa, a systematic scoping review from 2011 to 2021
Source: PLoS One. 2021 Nov 29;16(11):e0260411. doi: 10.1371/journal.pone.0260411 (PMC8629234; doi:10.1371/journal.pone.0260411)
Supplement: S1 File — (DOCX) [file pone.0260411.s002.docx]

MMAT Appraisal of individual studies

| **Part I: Mixed Methods Appraisal Tool (MMAT), version 2018** | | | | | | **1** | 2 | 3 | 4 | 5 | 6 | 7 | 8 | 9 | 10 |
| --- | --- | --- | --- | --- | --- | --- | --- | --- | --- | --- | --- | --- | --- | --- | --- |
| **Category of study designs** | **Methodological quality criteri** | **Responses** | | | | Sunitha et al. 2015 | Sahli et al. 2016 | Wentzel viljeon et al., 2017 | Gaziano et al. 2014 | Hendrik et al. 2014 | Hacking et al. 2016 | Rampamba et al. 2019 | Marfo et al. 2016 | van de Vijver et al., 2016 | Puoane et al., 2012 |
|  |  | **Yes** | **No** | **Can’t tell** | **Comments** |  |  |  |  |  |  |  |  |  |  |
| Quantitative randomized controlled trials | Is randomization appropriately performed? | √ |  |  |  |  |  |  |  |  |  | Yes |  |  |  |
|  | Are the groups comparable at baseline? | √ |  |  |  |  |  |  |  |  |  | Yes |  |  |  |
|  | Are there complete outcome data? | √ |  |  |  |  |  |  |  |  |  | Yes |  |  |  |
|  | Are outcome assessors blinded to the intervention provided? | √ |  |  |  |  |  |  |  |  |  | No |  |  |  |
|  | Did the participants adhere to the assigned intervention? | √ |  |  |  |  |  |  |  |  |  | Yes |  |  |  |
| Mixed methods | Is there an adequate rationale for using a mixed methods design to address the research question? | √ |  |  |  |  |  |  |  |  | Yes |  |  |  |  |
|  | Are the different components of the study effectively integrated to answer the research question? | √ |  |  |  |  |  |  |  |  | No |  |  |  |  |
|  | Are the outputs of the integration of qualitative and quantitative components adequately interpreted? | √ |  |  |  |  |  |  |  |  | Yes |  |  |  |  |
|  | Are divergences and inconsistencies between quantitative and qualitative results adequately addressed? | √ |  |  |  |  |  |  |  |  | Yes |  |  |  |  |
|  | Do the different components of the study adhere to the quality criteria of each tradition of the methods involved? | √ |  |  |  |  |  |  |  |  | Yes |  |  |  |  |
| Non-randomized studies | Are the participants representative of the target population? | √ |  |  |  | No | Yes | Yes | No | Yes |  |  | No | Yes | No |
|  | Are measurements appropriate regarding both the outcome and intervention (or exposure)? | √ |  |  |  | Yes | Yes | Yes | Yes | Yes |  |  | Yes | Yes | Yes |
|  | Are there complete outcome data? | √ |  |  |  | Yes | Yes | No | Yes | Yes |  |  | Yes | Yes | Yes |
|  | Are the confounders accounted for in the design and analysis? | √ |  |  |  | Yes | Yes | No | No | Yes |  |  | Yes | Yes | No |
|  | During the study period, is the intervention administered (or exposure occurred) as intended? | √ |  |  |  | Yes | Yes | Yes | Yes | Yes |  |  | Yes | Yes | No |
